# Functional signatures in Great Britain: A dataset

**DOI:** 10.1016/j.dib.2022.108335

**Published:** 2022-05-29

**Authors:** Krasen Samardzhiev, Martin Fleischmann, Daniel Arribas-Bel, Alessia Calafiore, Francisco Rowe

**Affiliations:** a Geographic Data Science Lab, University of Liverpool, Liverpool, L69 7ZX; b Alan Turing Institute, British Library, 96 Euston Road, London, NW1 2DB

**Keywords:** Geographic data science, Urban analytics, Functional areas, Spatial data, Land use

## Abstract

The spatial distribution of activities and agents within cities, conceptualised as an urban function, profoundly affects how different areas are perceived and lived. This dataset introduces the concept of functional signatures - contiguous areas of a similar urban function delineated based on enclosed tessellation cells (ETC) - and applies it to the area of Great Britain. ETCs are granular spatial units, which capture function based on interpolations from open data inputs stretching from remote sensing to land use, census and points of interest data. The spatial extent of each signature type is defined by grouping ETCs using cluster analysis, based on similarity between their functional profiles, inferred by the data linked to each cell. This approach results in a dataset that reflects urban function as a composite of aspects, rather than a singular use, and is built up from granular spatial units. Furthermore, the underlying data are sourced from available open data products, which together with a method and code fully available, yields a fully reproducible pipeline and makes our dataset and open data product.

Both the final classification composed of 17 types of functional signatures and the underlying data collected on the level of enclosed tessellation cells are included in the release and described in this report.

## Specifications Table

**Table:** 

Subject	Geography
Specific subject area	Urban geography, Geographic Data Science
Type of data	Table Spatial polygons
How the data were acquired	All data is secondary data, downloaded and processed as the Urban Grammar project [2]. Specifically, the datasets were downloaded from the ONS, UK Data service, Scottish statistics website, World pop website, English, Workplace census, Copernicus land use, Scottish and Welsh historical buildings and CDRC websites. The exact URLS are available in the Urban Grammar web page [2]. All data was processed using the GDS environment [3]
Data format	Raw Analyzed
Description of data collection	The functional data relating to cells was processed from the same sources and following Urban Grammar [2]. All data processing was done in Python within the GDS environment [3].The data was preprocessed and the functional signature clustering was carried out using K-Means. There were two main criteria for data selection. First the data had to be open sourced functional data, in order for the methodology to be reproducible. Second, the data had to cover the whole of Great Britain.
Data source location	Great Britain
Data accessibility	Repository name: Functional signatures Data identification number (permanent identifier, i.e. DOI number): https://doi.org/10.6084/m9.figshare.c.5906981.v1 Direct link to the dataset: https://figshare.com/collections/Functional_signatures/5906981/1

## Value of the Data

• This dataset provides information on the delineation of different types of functional areas across Great Britain. Specifically, it defines the spatial extent of functional signatures for the whole of GB, based on population, nightlights, workplace land use and amenity data. The signatures are clusters which represent distinct types of usage and human activities which occur within them. The raw data units used to create the clusters are small in scale, centred around urban structures, cover the whole of the UK and capture multiple aspects of functional usage.
• The data can be used by urban researchers interested in areas intersecting with functional usage - mobility, morphology, land use research and others.
• Both the unprocessed and clustered data can be linked with different types of mobility data, in order to gain insights into the multitude of ways people experience and access urban function at high spatial resolutions. Thus, the dataset can be useful for planners and policy making in the modelling of inequality, disease spread, accessibility to greenspaces, amenities, and cultural areas. Furthermore, the data can be paired up with street and building characteristics and environmental factors (such as air pollution) to understand what effect, if any, these have on functionality of the area and to study concepts such as urban vitality - the link between mixed usage and the prosperity of urban areas.
• For researchers interested in more granular or larger scales of functional usage, the enclosed tessellation cells, the final signatures and the proposed methodology can be used to accommodate that need.

## 1 Data Description

The open data product is composed of datasets reflecting different stages of delineation of functional signatures. The first file - tessalation_data.csv - contains all the data collected on the level of enclosed tessellation cells. The second and main file - functional_signatures.gpkg - contains the final signature geometry assigned into one of the 17 types.

Each row in the unprocessed dataset represents an enclosed tessellation cell in GB and its associated data. ETCs are small pieces of land constructed using Voronoi tessellation based on building footprint polygons bounded by roads, railways or natural barriers. An enclosed tessellation cell contains 50 types of characters described in the table below, reflected by 146 variables. Each character is represented in the dataset by three values - the quartiles (25%, 50%, 75%) of the distribution of that particular type of character in five levels of topological neighbours around the tessellation cell. This is done in order to capture the local spatial context of each tessellation cell. Accessibility variables that capture spatial context by design are associated with one value each. More information about the construction of the ETCs is presented in the next section.

In Table 1 the “Variable” column is the colloquial name of the variable and the “Description” column provides a description of the information the variable captures. The “input spatial unit” describes the spatial unit in the data source. Finally, “transfer method” shows how the data was interpolated to the tessellation cells. For in the first row, the input spatial unit is “Vector (output area polygon)” means that the population data is aggregated to UK census output area polygons. The transfer method is “Building-based dasymetric areal interpolation”, which means that the Output area data polygon data was interpolated to the buildings it encompases. As described in the next section, the input spatial units and the transfer methods both follow the methodology designed by Fleishmann and Arribas-Bel [2]. For the Corine land classification data, the descriptions come from the official website [8].

**Table 1:** Characters data in enclosed tessellation cells.

Variable	Description	Input spatial unit	Transfer method
Population	National and subnational mid-year population estimates for the UK and its constituent countries by administrative area. Source: ONS Census Output Area population estimates, Statistics.gov.scot	Vector (output area polygon)	Building-based dasymetric areal interpolation
Night lights	Nightlight intensity Source: VIIRS DNB Nighttime Lights	Raster (500m)	Zonal statistics
Workplace population [Agriculture, energy and water]	Number of people working in Agriculture, energy and water sectors as defined by the 2011 census: Industry hierarchy Source: ONS Census Workplace population, Scotland's census Workplace population	Vector (output area polygon)	Building-based dasymetric areal interpolation
Workplace population [Manufacturing]	Number of people working in Manufacturing as defined by the 2011 census: Industry hierarchy Source: ONS Census Workplace population, Scotland's census Workplace population	Vector (output area polygon)	Building-based dasymetric areal interpolation
Workplace population [Construction]	Number of people working in Construction as defined by the 2011 census: Industry hierarchy Source: ONS Census Workplace population, Scotland's census Workplace population	Vector (output area polygon)	Building-based dasymetric areal interpolation
Workplace population [Distribution, hotels and restaurants]	Number of people working in Distribution, hotels and restaurants as defineb by the2011 census: Industry hierarchy Source: ONS Census Workplace population, Scotland's census Workplace population	Vector (output area polygon)	Building-based dasymetric areal interpolation
Workplace population [Transport and communication]	Number of people working in Transport and communication] as defined by the 2011 census: Industry hierarchy Source: ONS Census Workplace population, Scotland's census Workplace population	Vector (output area polygon)	Building-based dasymetric areal interpolation
Workplace population [Financial, real estate, professional and administrative activities]	Number of people working in Financial, real estate, professional and administrative activities as defined by the 2011 census: Industry hierarchy Source: ONS Census Workplace population, Scotland's census Workplace population	Vector (output area polygon)	Building-based dasymetric areal interpolation
Workplace population [Public administration, education and health]	Number of people working in Public administration, education and health as defined by the 2011 census: Industry hierarchy Source: ONS Census Workplace population, Scotland's census Workplace population	Vector (output area polygon)	Building-based dasymetric areal interpolation
Workplace population [Other]	Number of people working in Other sectors as defined by the 2011 census: Industry hierarchy Source: ONS Census Workplace population, Scotland's census Workplace population	Vector (output area polygon)	Building-based dasymetric areal interpolation
Land cover [Airports]	Source: Copernicus Land Monitoring Service Airports installations: runways, buildings and associated land. This class is assigned for any kind of ground facilities that serve airborne transportation.	Vector (land cover zone polygon)	Areal interpolation
Land cover [Non-irrigated arable land]	Source: Copernicus Land Monitoring Service Cultivated land parcels under rainfed agricultural use for annually harvested non-permanent crops, normally under a crop rotation system, including fallow lands within such crop rotation. Fields with sporadic sprinkler-irrigation with non-permanent devices to support dominant rainfed cultivation are included.	Vector (land cover zone polygon)	Areal interpolation
Land cover [Industrial or commercial units]	Source: Copernicus Land Monitoring Service Buildings, other built-up structures and artificial surfaces (with concrete, asphalt, tarmacadam, or stabilised like e.g. beaten earth) occupy most of the area. It can also contain vegetation (most likely grass) or other non-sealed surfaces. This class is assigned for land units that are under industrial or commercial use or serve for public service facilities.	Vector (land cover zone polygon)	Areal interpolation
Land cover [Salt marshes]	Source: Copernicus Land Monitoring Service Vegetated low-lying areas in the coastal zone, above the high-tide line, susceptible to flooding by seawater. Often in the process of being filled in by coastal mud and sand sediments, gradually being colonized by halophilic plants.	Vector (land cover zone polygon)	Areal interpolation
Land cover [Estuaries]	Source: Copernicus Land Monitoring Service The mouth of a river under tidal influence within which the tide ebbs and flows.	Vector (land cover zone polygon)	Areal interpolation
Land cover [Sport and leisure facilities]	Source: Copernicus Land Monitoring Service This class is assigned for areas used for sports, leisure and recreation purposes. Camping grounds, sports grounds, leisure parks, golf courses, racecourses etc. belong to this class, as well as formal parks not surrounded by urban areas.	Vector (land cover zone polygon)	Areal interpolation
Land cover [Green urban areas]	Source: Copernicus Land Monitoring Service Areas with vegetation within or partly embraced by urban fabric. This class is assigned for urban greenery, which usually has recreational or ornamental character and is usually accessible for the public.	Vector (land cover zone polygon)	Areal interpolation
Land cover [Discontinuous urban fabric]	Source: Copernicus Land Monitoring Service The discontinuous urban fabric class is assigned when urban structures and transport networks associated with vegetated areas and bare surfaces are present and occupy significant surfaces in a discontinuous spatial pattern. The impermeable features like buildings, roads and artificially surfaced areas range from 30 to 80 % land coverage.	Vector (land cover zone polygon)	Areal interpolation
Land cover [Pastures]	Source: Copernicus Land Monitoring Service Permanent grassland characterized by agricultural use or strong human disturbance. Floral composition dominated by graminacea and influenced by human activity. Typically used for grazing-pastures, or mechanical harvesting of grass–meadows.	Vector (land cover zone polygon)	Areal interpolation
Land cover [Broad-leaved forest]	Source: Copernicus Land Monitoring Service Vegetation formation composed principally of trees, including shrub and bush understorey, where broad-leaved species predominate.	Vector (land cover zone polygon)	Areal interpolation
Land cover [Mineral extraction sites]	Source: Copernicus Land Monitoring Service Open-pit extraction sites of construction materials (sandpits, quarries) or other minerals (open-cast mines). Includes flooded mining pits.	Vector (land cover zone polygon)	Areal interpolation
Land cover [Port areas]	Source: Copernicus Land Monitoring Service Infrastructure of port areas (land and water surface), including quays, dockyards and marinas.	Vector (land cover zone polygon)	Areal interpolation
Land cover [Road and rail networks and associated land]	Source: Copernicus Land Monitoring Service Motorways and railways, including associated installations (stations, platforms, embankments, linear greenery narrower than 100 m). Minimum width for inclusion: 100 m.	Vector (land cover zone polygon)	Areal interpolation
Land cover [Water bodies]	Source: Copernicus Land Monitoring Service Natural or artificial water bodies with presence of standing water surface during most of the year.	Vector (land cover zone polygon)	Areal interpolation
Land cover [Land principally occupied by agriculture, with significant areas of natural vegetation]	Source: Copernicus Land Monitoring Service Areas principally occupied by agriculture, interspersed with significant natural or semi-natural areas (including forests, shrubs, wetlands, water bodies, mineral outcrops) in a mosaic pattern.	Vector (land cover zone polygon)	Areal interpolation
Land cover [Mixed forest]	Source: Copernicus Land Monitoring Service Vegetation formation composed principally of trees, including shrub and bush understorey, where neither broad-leaved nor coniferous species predominate.	Vector (land cover zone polygon)	Areal interpolation
Land cover [Peat bogs]	Source: Copernicus Land Monitoring Service Wetlands with accumulation of considerable amount of decomposed moss (mostly Sphagnum) and vegetation matter. Both natural and exploited peat bogs.	Vector (land cover zone polygon)	Areal interpolation
Land cover [Natural grasslands]	Source: Copernicus Land Monitoring Service Grasslands under no or moderate human influence. Low productivity grasslands. Often situated in areas of rough, uneven ground, steep slopes; frequently including rocky areas or patches of other (semi-)natural vegetation.	Vector (land cover zone polygon)	Areal interpolation
Land cover [Moors and heathland]	Source: Copernicus Land Monitoring Service Vegetation with low and closed cover, dominated by bushes, shrubs, dwarf shrubs (heather, briars, broom, gorse, laburnum etc.) and herbaceous plants, forming a climax stage of development.	Vector (land cover zone polygon)	Areal interpolation
Land cover [Transitional woodland-shrub]	Source: Copernicus Land Monitoring Service Transitional bushy and herbaceous vegetation with occasional scattered trees. Can represent woodland degradation, forest regeneration / recolonization or natural succession.	Vector (land cover zone polygon)	Areal interpolation
Land cover [Continuous urban fabric]	Source: Copernicus Land Monitoring Service The continuous urban fabric class is assigned when urban structures and transport networks are dominating the surface area. > 80% of the land surface is covered by impermeable features like buildings, roads and artificially surfaced areas. Non-linear areas of vegetation and bare soil are exceptional.	Vector (land cover zone polygon)	Areal interpolation
Land cover [Intertidal flats]	Source: Copernicus Land Monitoring Service Coastal zone under tidal influence between open sea and land, which is flooded by sea water regularly twice a day in a ca. 12 hours cycle. Area between the average lowest and highest sea water level at low tide and high tide. Generally non-vegetated expanses of mud, sand or rock lying between high and low water marks.	Vector (land cover zone polygon)	Areal interpolation
Land cover [Sea and ocean]	Source: Copernicus Land Monitoring Service Zone seaward of the lowest tide limit.	Vector (land cover zone polygon)	Areal interpolation
Land cover [Construction sites]	Source: Copernicus Land Monitoring Service Spaces under construction development, soil or bedrock excavations, earthworks. This class is assigned for areas where landscape is affected by human activities, changed or modified into artificial surfaces, being in a state of anthropogenic transition.	Vector (land cover zone polygon)	Areal interpolation
Land cover [Burnt areas]	Source: Copernicus Land Monitoring Service Natural woody vegetation affected by recent fires.	Vector (land cover zone polygon)	Areal interpolation
Land cover [Dump sites]	Source: Copernicus Land Monitoring Service Public, industrial or mine dump sites.	Vector (land cover zone polygon)	Areal interpolation
Land cover [Complex cultivation patterns]	Source: Copernicus Land Monitoring Service Mosaic of small cultivated land parcels with different cultivation types -annual crops, pasture and/or permanent crops-, eventually with scattered houses or gardens.	Vector (land cover zone polygon)	Areal interpolation
Land cover [Inland marshes]	Source: Copernicus Land Monitoring Service Low-lying land usually flooded in winter, and with ground more or less saturated by fresh water all year round.	Vector (land cover zone polygon)	Areal interpolation
Land cover [Water courses]	Source: Copernicus Land Monitoring Service Natural or artificial water-courses serving as water drainage channels. Includes canals. Minimum width for inclusion: 100 m.	Vector (land cover zone polygon)	Areal interpolation
Land cover [Coniferous forest]	Source: Copernicus Land Monitoring Service Vegetation formation composed principally of trees, including shrub and bush understorey, where coniferous species predominate.	Vector (land cover zone polygon)	Areal interpolation
Land cover [Bare rocks]	Source: Copernicus Land Monitoring Service Scree, cliffs, rock outcrops, including areas of active erosion, rocks and reef flats situated above the high-water mark, inland salt planes.	Vector (land cover zone polygon)	Areal interpolation
Land cover [Coastal lagoons]	Source: Copernicus Land Monitoring Service Stretches of salt or brackish water in coastal areas which are separated from the sea by a tongue of land or other similar topography. These water bodies can be connected to the sea at limited points, either permanently or for parts of the year only.	Vector (land cover zone polygon)	Areal interpolation
Land cover [Beaches, dunes, sands]	Source: Copernicus Land Monitoring Service Natural non-vegetated expanses of sand or pebble/gravel, in coastal or continental locations, like beaches, dunes, gravel pads; including beds of stream channels with torrential regime. Vegetation covers maximum 10%.	Vector (land cover zone polygon)	Areal interpolation
Land cover [Agro-forestry areas]	Source: Copernicus Land Monitoring Service Annual crops or grazing land under the wooded cover of forestry species.	Vector (land cover zone polygon)	Areal interpolation
Land cover [Sparsely vegetated areas]	Source: Copernicus Land Monitoring Service Areas with sparse vegetation, covering 10-50% of surface. Includes steppes, tundra, lichen heath, badlands, karstic areas and scattered high-altitude vegetation.	Vector (land cover zone polygon)	Areal interpolation
Land cover [Fruit trees and berry plantations]	Source: Copernicus Land Monitoring Service Cultivated parcels planted with fruit trees and shrubs, intended for fruit production, including nuts. The planting pattern can be by single or mixed fruit species, both in association with permanently grassy surfaces.	Vector (land cover zone polygon)	Areal interpolation
NDVI	Normalised Difference Vegetation Index derived from Sentinel 2 cloud -free mosaic	Raster (10m)	Zonal statistics
Retail centres [distance to nearest]	Distance to closest Geolytix retail point	Vector (retail centre polygon)	Euclidean accessibility
Supermarkets [counts within 1200m]	Number of supermarkets within 1200m of the cell	Vector (point)	Network-constrained accessibility
Listed buildings [counts within 1200m]	Number of listed buildings within 1200m of the cell	Vector (point)	Network-constrained accessibility
FHRS points [counts within 1200m]	Number of Food hygiene rating scheme companies (bars, restaurants, takeouts, etc.) within 1200m of the cell	Vector (point)	Network-constrained accessibility
Cultural venues [counts within 1200m]	Number of cultural venues within 1200m of the cell	Vector (point)	Network-constrained accessibility

In addition to the variables, there is a hindex column, which uniquely identifies each row and can be used to link the rows with their corresponding spatial polygons produced by Fleishmann and Arribas-Bel [1].

The table describes the data associated with each tessellation cell in the enclosed tessellation cells table. Each row represents a character type - the first column is the name, while the second is a description of the information the variable captures. Columns three and four describe how the data was interpolated to the tessellation cell level, following the methodology used in [1].

**Fig. 1:**
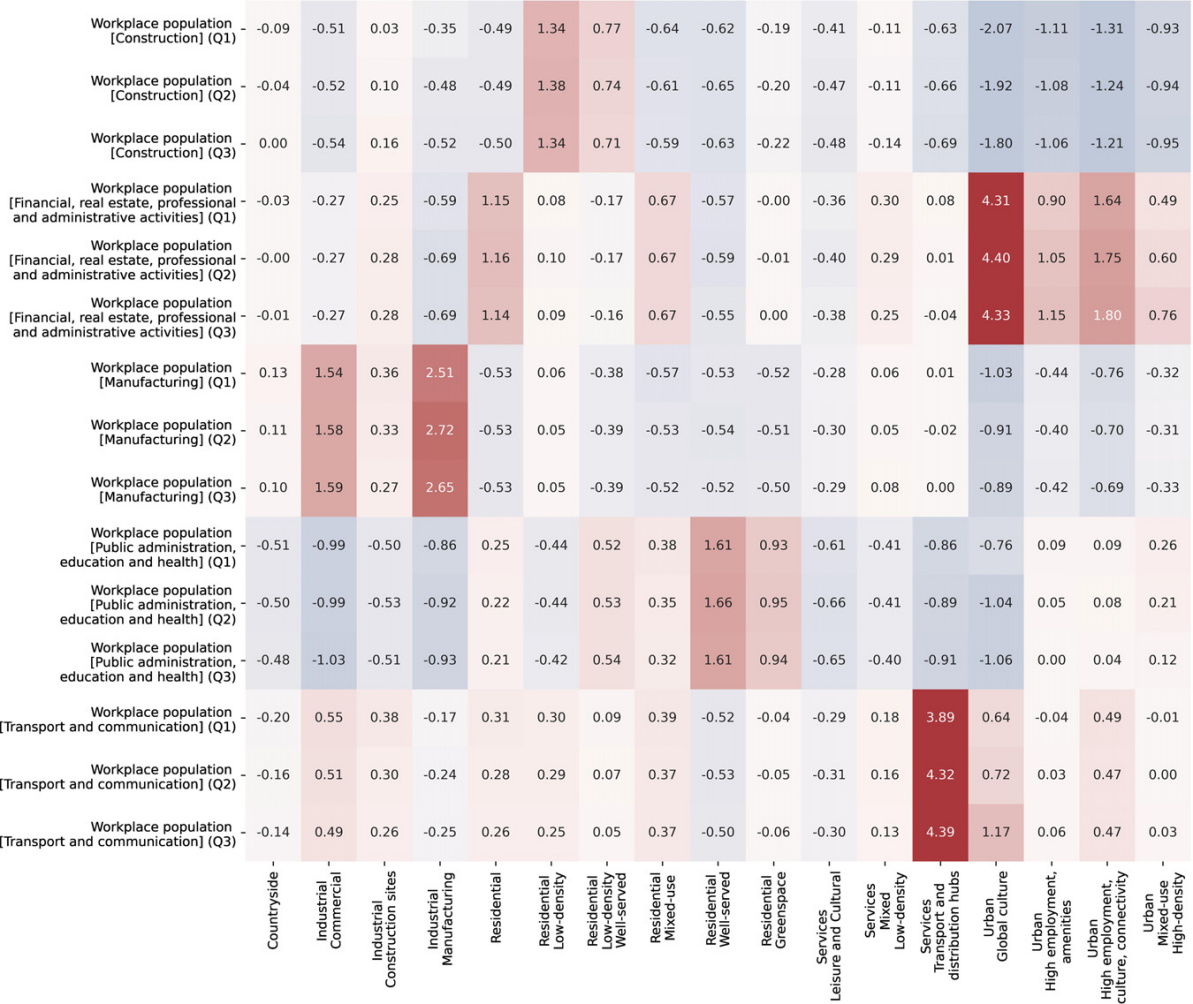
Workplace population heat map.

**Fig. 2:**
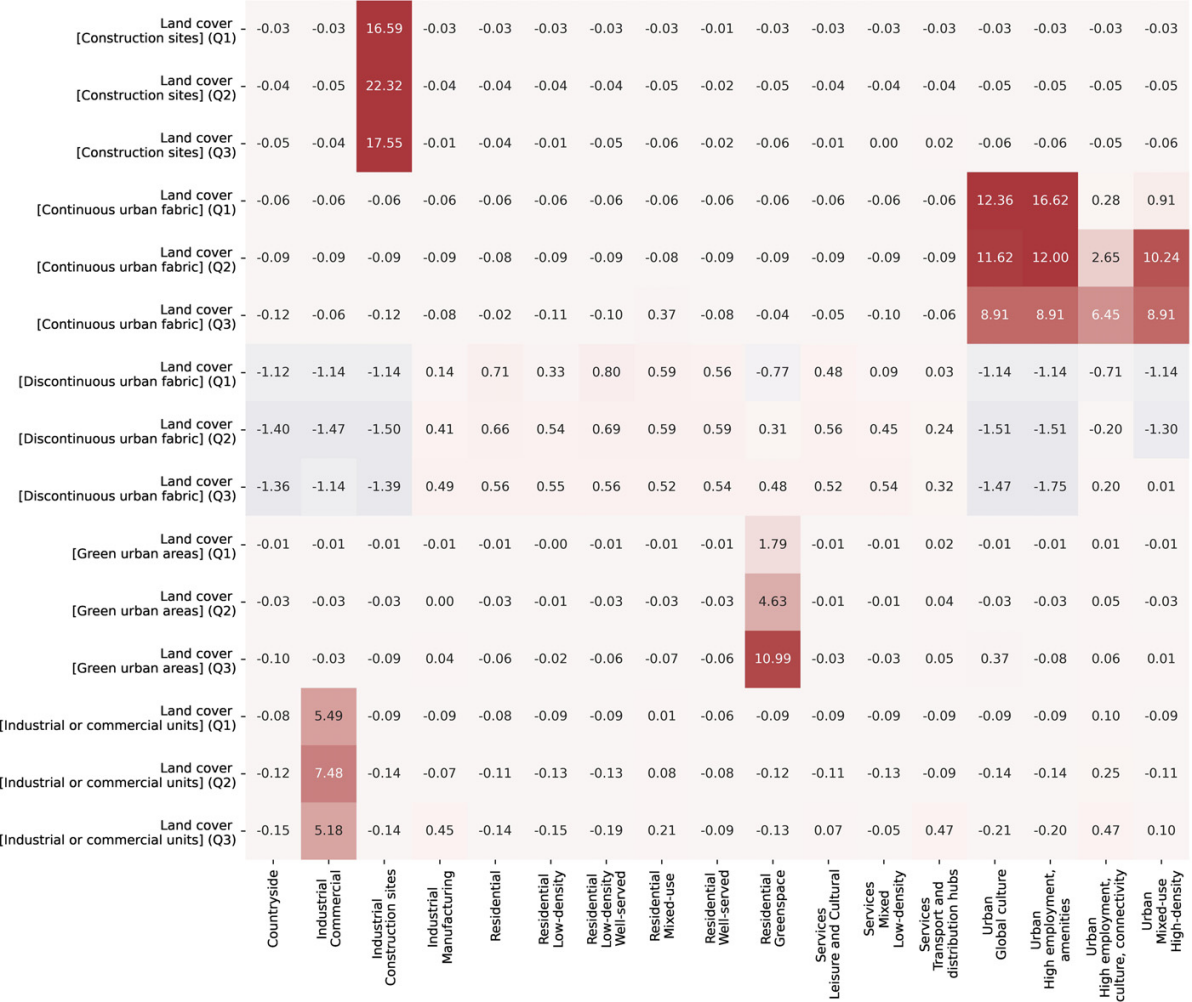
Land use attributes heatmap.

**Fig. 3:**
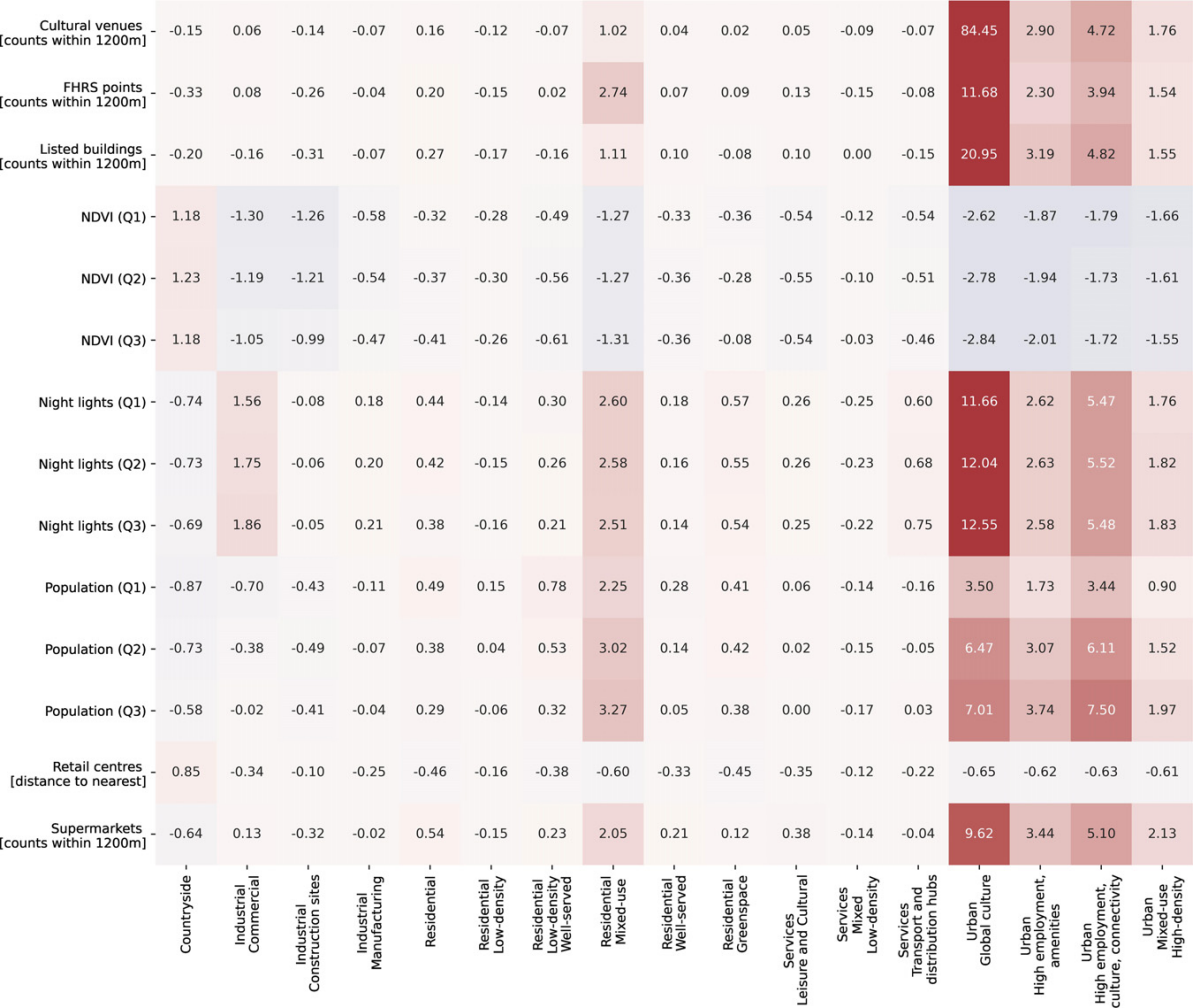
Other attributes heatmap.

**Fig. 4:**
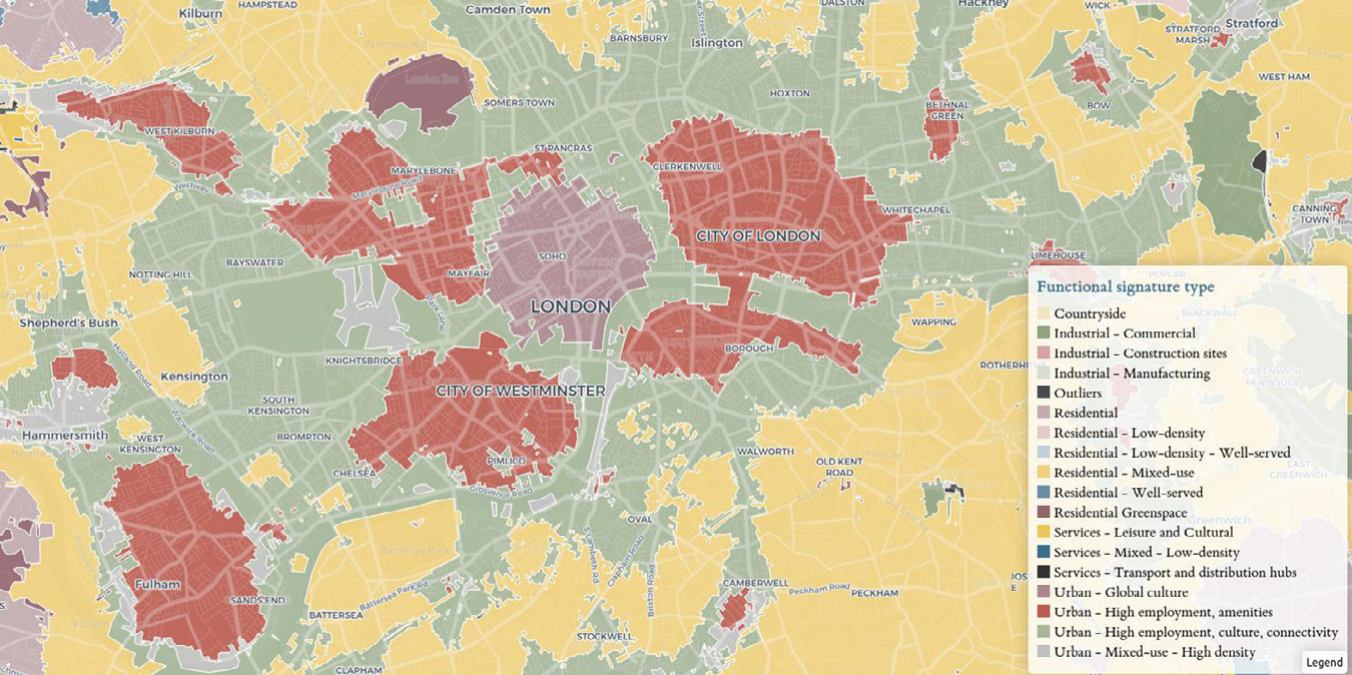
Functional signatures in Central London.

Table 2 describes the final functional signature types and provides a description of what each identified signature type represents. The last column shows the number of tessellation cells within the cluster. The signature class descriptions are derived from the distributions of variables within each cluster. Three heatmaps with subsets of the 146 variables are shown in Figs. 1–3. The values within the cells are the standard deviations of the corresponding variable across the whole dataset. Relatively higher values are in red, while relatively lower values are in blue. Fig. 1 represents the fifteen variables from the Workplace census with the most variability across the clusters, Fig. 2 - from the Land use variables and Fig. 3 from the other types of data. Together the three figures describe the 45 most important variables used in the naming and description of each functional signature. The “Outliers” signature type was excluded from these comparisons since it skews the variable selection and values. The clustering and naming methodology are specified in detail in the next section. Fig. 4 below shows an example map of functional signatures within Central London.

**Table 2:** Functional Signatures.

Custer Family	Functional Signature	Description	Number of tessellation cells in cluster
*Residential*	*Residential - Low-density*	This signature encomapses the areas around cities as well as small towns, with a predominantly residential focus exemplified by the high value of discontinuous land use compared to other variables in the cluster. In contrast to 'Residential - Low-density - Highly-served' this area has more transport links.	1,755,731
*Residential*	*Residential - Low-density - Well-served*	Residential areas on the peripheries of cities with less nightlights and population than other residential classes and a relatively high-number of construction projects and public services to "Residential areas - low-density".	1,608,363
*Services*	*Services - Mixed - Low-density*	This class primarily contains areas outside of urban centres or near small cities which have multiple functional uses. In contrast to the 'Services - Leisure and Cultural' this class has access to proportionally more professional, administrative and other services and less population.	1,510,671
*Residential*	*Residential*	This class is the base residential type among all the clusters. It encompanses areas with a primarily residential usage in urban areas. The class includes - housing developments, council estates, residential neighbourhoods and areas with many apartment complexes.	1,253,444
*Residential*	*Residential - Well-served*	Urban areas with a primarily residential focus. Compared to other residential areas, these have access to greenspace and nearby public services such as hospitals and schools. They are not necessarily richer than other residential areas.	2,144,424
*Services*	*Services - Leisure and Cultural*	Areas with a focus on leisure, tourism and culture with a high number of restaurants, hotels and activities available and a high proportion of workplace population in these sectors. This class includes places such as malls, golf clubs, seaside towns and others.	773,590
*Industrial*	*Industrial - Manufacturing*	Industrial, manufacturing and distribution sites typically outside cities. This signature type has an extremely high value of industrial land use compared to other signatures.	748,678
*Industrial*	*Industrial - Construction sites*	Large areas under construction, predominantly outside cities.This signature type has an extremely high value of construction land use compared to other signatures.	25,105
*Countryside*	*Countryside*	This cluster encompases both accesable and innacessable countryside, as well as agricultural land. This is the only class of functional signatures with land use types such as orchards, pastures, on the one hand and bogs and mountains on the other.	3,819,218
*Residential*	*Residential - Mixed-use*	Urban areas with a mixed residential and work usage near city centres. The cluster encompases areas with high levels of population, less houses and more access to jobs, offices and services.	318,464
*Services*	*Services - Transport and distribution hubs*	Important places in the transport and distribution network such as airports, train depos and distribution centres. This signature type has high proportions of people working in the transport and distribution sector, and higher levels of airport and rail land use values.	165,298
*Industrial*	*Industrial - Commercial*	Industrial areas with a primarily commercial focus, typically inside cities. This signature type has an extremely high value of industrial land use compared to other signatures. In contrast to “Industrial - Manufacturing” it is closer to retail centres and cultural avenues.	204,564
*Urban*	*Urban - High employment, culture, connectivity*	Urban areas which encompass different functionalities, reflected in the high number of professional offices, listed buildings, cultural venues and transportation links.	37,073
*Residential Greenspace*	*Residential Greenspace*	Urban greenspace areas such as parks and large gardens. This signature type has an extremely high value of urban green space land use compared to other signatures.	70,361
*Urban*	*Urban - Mixed-use - High density*	Urban areas with a mixed residential and work usage near city centres. In contrast to 'Residential - Mixed-use', this cluster has higher available services, access to jobs and cultural venues and is less residential.	53,877
*Urban*	*Urban - High employment, amenities*	This class consists of areas with high population, a large number of professional services and numerous amenities. The specific focus of the areas within it can be different - financial, retail or other. In contrast to “Urban - High employment, culture, connectivity” it has a higher proportion of people working in retail and food sector, but less in transport and distribution.	49,729
*Urban*	*Urban - Global culture*	This cluster encompasses only one area in the UK - Soho in London. It is characterised by extremely high access to cultural venues, amenities, as well as high access to services.	603
*Outliers*	*Outliers*	Tessellation cells which do not fall within the other clusters and do not have a clear dominant functional usage.	385

This table presents final functional signature types. The first column identifies the cluster class, the second one the full cluster name. The “Description” column presents a short description of the signature type, while the last column shows the number of enclosed tessellation cells in that cluster.

This heatmap shows the distribution of workplace population variables within each signature. The relatively higher values are shown in red, while the relatively lower values are shown in blue. The cell values represent the mean standard deviations for a particular cluster variable pair, calculated across the whole dataset.

This heatmap shows the distribution of land use variables within each signature. The relatively higher values are highlighted in red, while the relatively lower values are shown in blue. The cell values represent the mean standard deviations for a particular cluster variable pair, calculated across the whole dataset.

This heatmap shows the distribution of variables which are not workplace population or land use. The relatively higher values are highlighted in red, while the relatively lower values are shown in blue. The cell values represent the mean standard deviations for a particular cluster variable pair, calculated across the whole dataset.

A map of the spatial distribution of signature types centred around Central London.

**Fig. 5:**
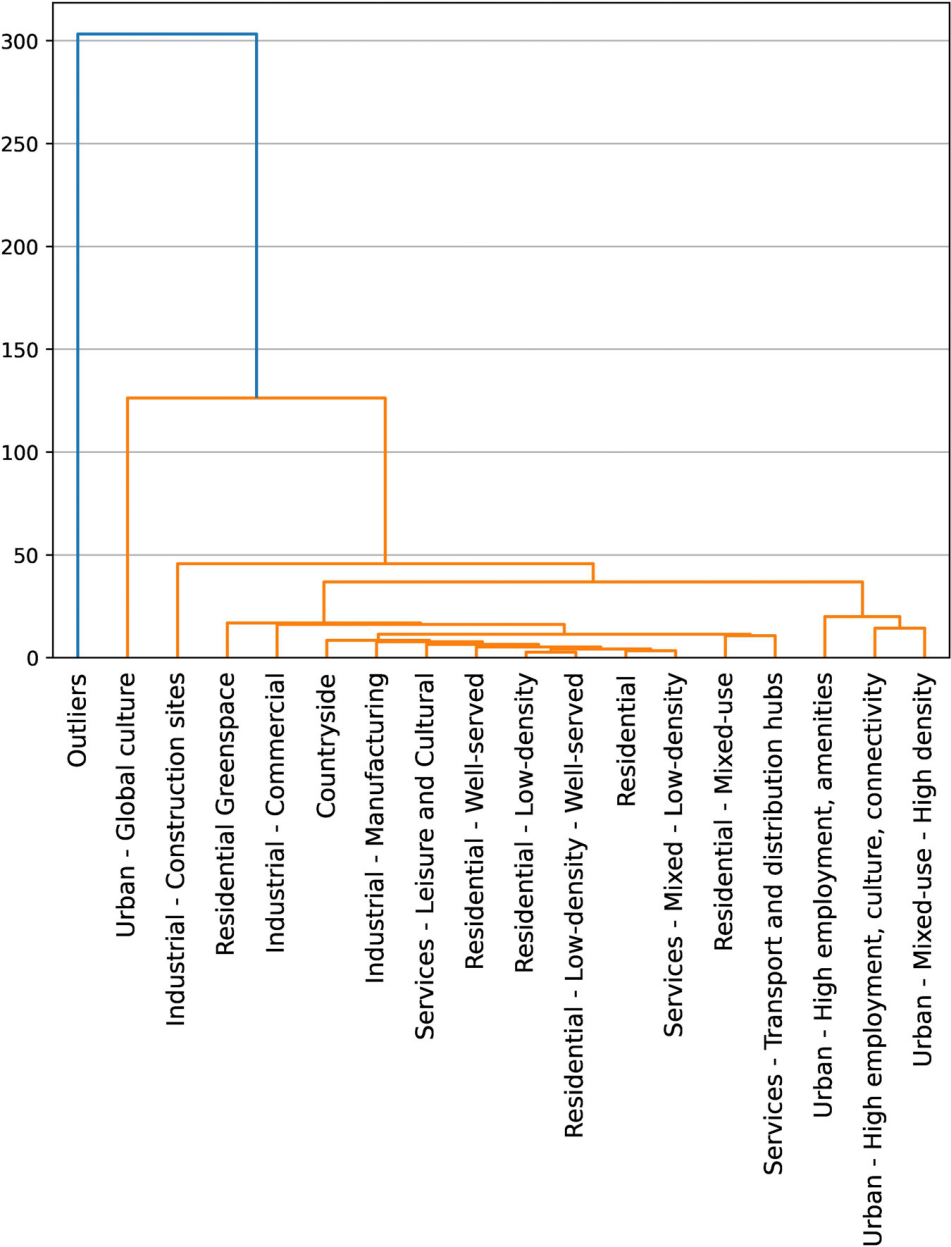
Hierarchical relationship between signatures.

Fig. 5 shows the hierarchical relationship between the clusters, derived using Ward's hierarchical clustering. Clusters which are connected at a lower distance are on average more similar than those connected at higher distances. This is not the clustering methodology used in the paper and is done to demonstrate the relationship between the already processed clusters.

Distances between the final signatures, derived using ward single-linkage clustering.

## 2 Experimental Design, Materials and Methods

### 2.1 Data

The functional data collected on the level of enclosed tessellation cells are derived from the open data product published by Fleischmann and Arribas-Bel who generated the input but focused on a combination of function and form into a singular classification [1]. The data product presented in this paper deals only with one component of the original dataset - urban function and uses the same functional characters and geometries for the tessellation cells. The whole methodology with reproducible code and datasets is available in [2]. We present a summary of the methodology below.

The core unit of analysis is the enclosed tessellation cell (ETC). The first step in the creation of ETCs is to split the GB into enclosures - non-overlapping contiguous areas based on barriers such as roads, railways, and water bodies. ETC are then created within each enclosure as a polygon-based Voronoi tessellation based on building footprints, using the buildings within each of these areas as anchors [1]. The resulting Voronoi cells are used as a spatial unit to which all functional data is attached.

The second step consists of interpolating the functional data described in Table 1 to the cells.

This is done in two ways: the data is either interpolated based on zonal statistics or in the case of the accessibility variables based on counts or nearest distances. Lastly, in order to capture the relevant spatial context around the cells, a neighbourhood around each cell is defined based on its five nearest topological neighbours. Within each cell, we measure the first, second and third quartile of distribution of each character weighted by the inverse distance between the cell of origin and its neighbours. It should be noted that this is slightly different from the procedure used in Fleischmann and Arribas-Bel [1,2], where the neighbourhood is defined by ten topological neighbours. This was done in order to derive a finer grained functional classification. For the whole of the GB this results in ∼14 million cells with 146 variables, from the 50 types of variables described in the previous section.

These tessellation cells and their associated data were clustered to generate the functional areas.

The only processing applied to the tessellation cell data, before the clustering, is as follows: First, the workplace values were normalised, for each cell, in order to focus the comparisons on the distribution of types of jobs and not their absolute values. This does not adversely affect the dataset, since population effects were still captured by other variables such as total population. Next, the associated NDVI variables were transformed to be more comparable to the other land use measures. The index was shifted to the positive dimension only, by adding the dataset minimum value to all variables. As a last step all the data was standardised, in order to account for scale effects during the clustering.

### 2.2 Methodology

In order to derive the final functional area delineations, the data is clustered in three stages.

The first stage partitioned the whole dataset, the second stage clustered individual clusters from stage one separately and the third stage - specific second stage clusters. Three clustering runs were necessary in order to break down the data in separate functional areas at smaller scales. Furthermore, different types of variables were dominant at different iterations - in the first iteration the differences between clusters were mostly due to differences in land use variables, in the second and third the differences were dominated by workplace population and other characteristics. Therefore, in order to capture the differences across all 50 variable types, three iterations were needed.

The goal of the clustering is to detect areas with different functional characteristics. During the clustering itself several intermediate or final clusters were assigned to other already defined signature types. This was done to get a more compact set of final clusters. For example, intermediate clusters, which represent greenspaces of different sizes were grouped together. Similarly, some detected areas such as airports, were added to a more general cluster - transport hubs - due to their small cluster size. In total this is done for six clusters which represent less than 15,000 tessellation cells out of 14 million. These areas can be separated with further processing if needed by researchers. The analysis terminated after the third stage, since subclustering further only highlighted differences in magnitudes and gradations of already existing clusters.

The specific clustering methodology used throughout the paper is mini batch k-means. This algorithm partitions the data so as to minimise the sum of squares differences between the data in the different clusters. The methodology was chosen, since it can cope with the large size of the data and the high dimensionality. The parameters used for each run were held constant for all clustering runs:

- 100 random centre initialisations,
- a Batch size of 1 million observations,
- maximum number of iterations - 1000
- Random state - 42,
- different values for the choice of clusters - K - was explored and a number was chosen based on the quality of the clustering.

The clustergram was the main tool used to identify the optimal number of clusters (K) at each stage. A clustergram is a tool which visually shows the relative sizes of clusters as well as their relative separation based on the weighted first principal component of values within [7]. Most importantly, it also shows how points move between clusters as K increases. In Fig. 5, the thickness of the lines represents the number of points moving from one cluster to another while the size of the points represents the sizes of the clusters. The Y axis is the weighted first principal component and the X axis is the number of clusters. Values of K which resulted in larger distance between clusters and non-skewed cluster splits were preferred. Several other methods were used to evaluate the potential clusters. First, there was a qualitative exploration of the delineated signatures within the Liverpool area using local knowledge and second, the distribution of variables within clusters was analysed using heat maps such as in Fig. 2. In addition to the clustergram, qualitative validation and the heatmaps, three additional metrics were used - silhouette score [5], Davies-Bouldin [4] score, and Calinski-Harabasz index [6]. The silhouette score measures the overlap between clusters based on the distances between points, their assigned clusters and their closest clusters. Similarly, the Davies-Bouldin score is defined as the average similarity measure of each cluster with its most similar cluster, where similarity is the ratio of within-cluster distances to between-cluster distances and lower values mean more compact and distant clusters. Lastly, the Calinski-Harabasz index is the ratio of the sum of between-clusters dispersion and of within-cluster dispersion for all clusters, where a higher Calinski-Harabasz score relates to a model with better defined clusters.

Once all tessellation cells are assigned to clusters and the clustering procedure finishes, all contiguous tessellation cells assigned to the same cluster are spatially merged together into a single area, in order to derive the final functional signatures.

### 2.3 Clustering results

**Fig. 6:**
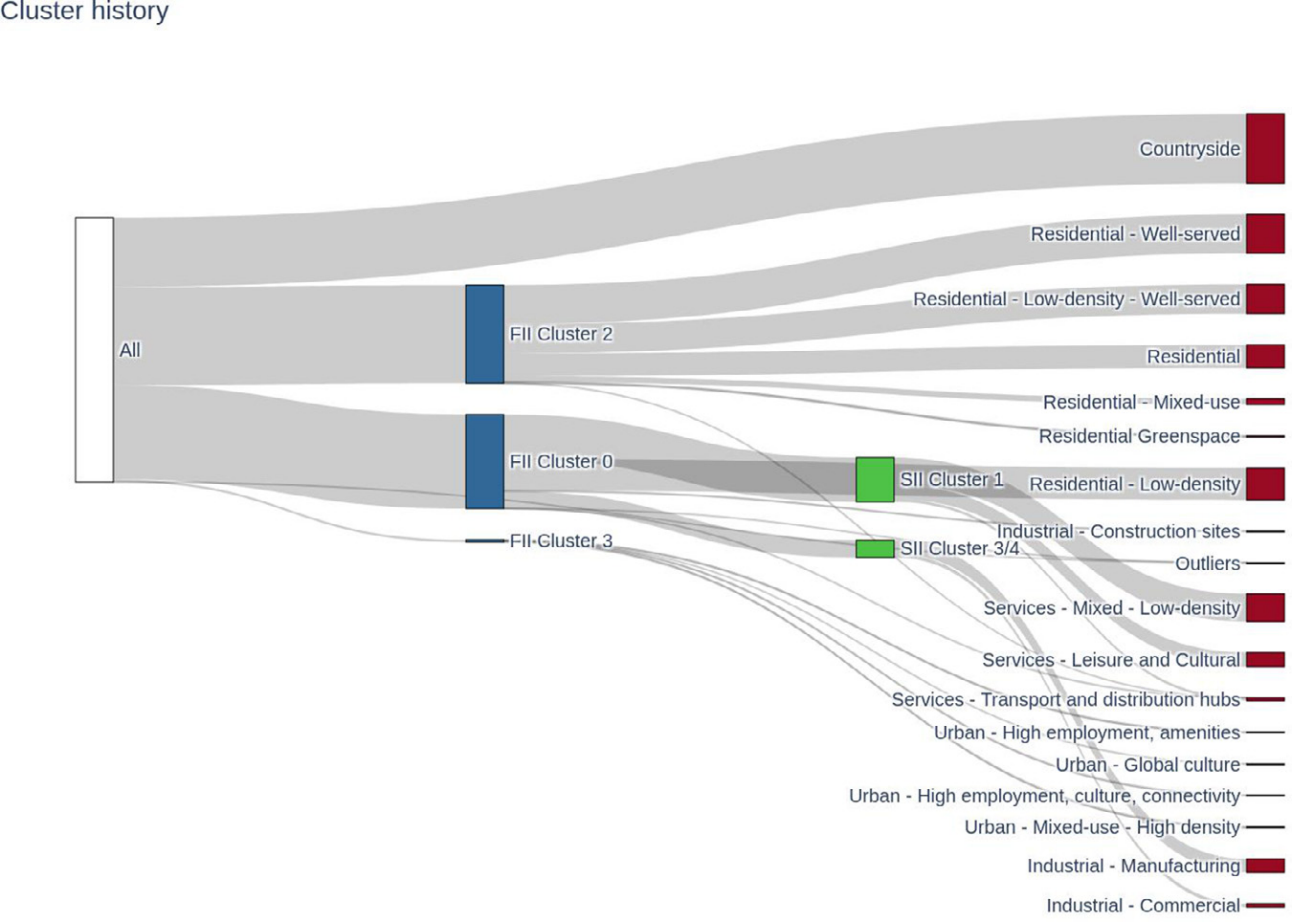
Cluster history.

The whole clustering procedure took three iterations to finish. The relative size of the clusters and how they are obtained is shown in Fig. 6. The red colour nodes are the final clusters, the white node is the whole dataset, the blue nodes are the intermediate clusters obtained from the first clustering and the green ones are the second intermediate clusters. FII stands for “First Iteration Intermediate” while SII stands for “Second Iteration Intermediate”.

A Sankey Diagram which shows how points were assigned to different clusters, throughout the clustering process.

**Fig. 7:**
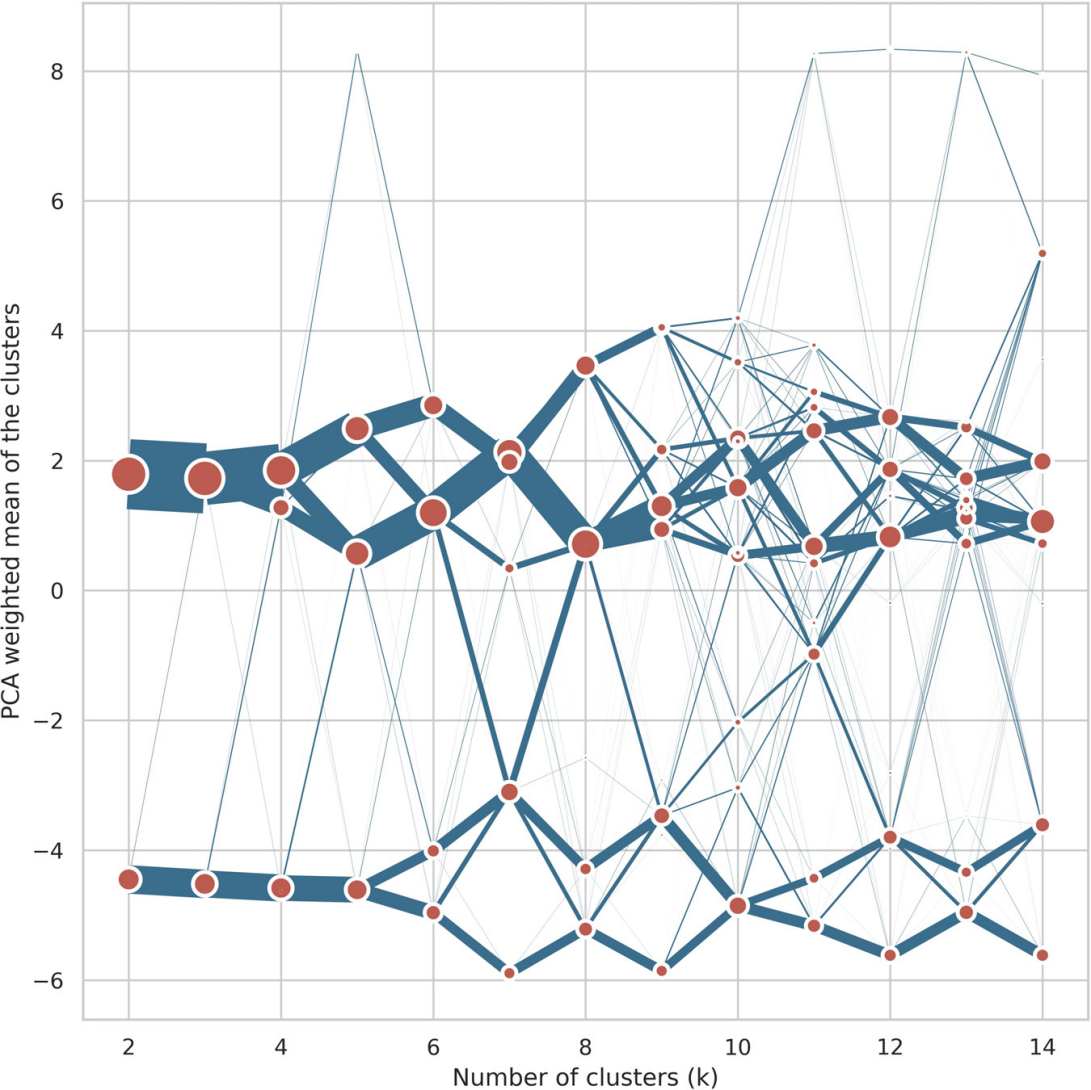
Clustergram for the first iteration of clustering.

For the initial clustering all values k ranging from 2-15 were explored. The corresponding clustergram is shown in Fig. 7, from it it can be seen that there are at least two well-separated groups in the data. However, values lower than 5 resulted in uneven clusters, while values larger than 5 resulted in clusterings with many outliers but similar cluster distributions to k = 5 for the majority of the data. Similarly, the choices of K with optimal Calinski-Harabasz, silhouette and Davies-Bouldin scores produced many outliers, skewed and non-differentiable signature types. K=5 showed large separation in the clustergram and resulted in balanced clusters with distinct heatmap distributions and was chosen for the first level clustering. Three of these clusters - 0,2,3 - are further processed and broken down in the next iteration. The other two clusters - 1, 4 - represent the final Outliers and Countryside clusters, which were not processed further.

This figure shows the relative separation and size of clusters for different values of K. The position of the clusters on the Y axis is based on the mean weighted first principle component of the dataset.

**Fig. 8:**
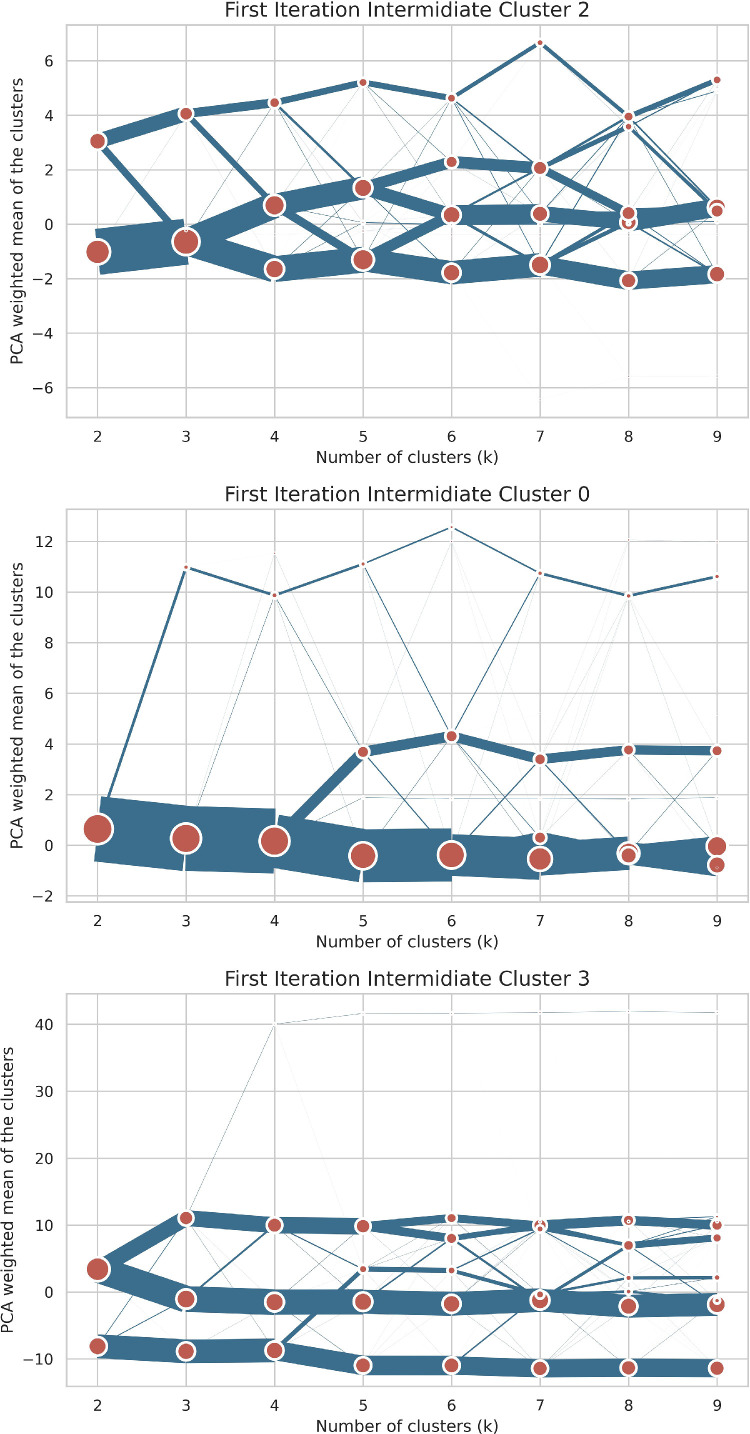
Clustergrams for the second iteration of clustering.

In the second iteration each level one cluster is clustered individually. This means that there were 3 additional independent clusterings. All sets of cluster runs followed the initial methodology and parameter choices laid out above. Similarly, to the previous iteration all optimal values based on the metrics were analysed and tested. The clustergrams for this iteration are shown in Fig. 8.

Each row shows the relative separation and size of clusters for different values of K, for specific clusters from the first clustering iteration. The position of the clusters on the Y axis is based on the mean weighted first principle component of the dataset.

The first clustering processed the “ First iteration intermediate cluster 2”, “FII Cluster 2” in Fig. 6 and the first row in Fig. 8. The choice of K was driven by similar considerations as in the first stage - good separation on the clustergram, qualitative soundness and the second best Davies-Bouldin score. The final choice of K was seven clusters. The first of which form six of the residential clusters described in Table 2 and have cluster family “Residential” prepended to their names. The seventh cluster represents parts of airports near residential areas, as evidenced by the relatively high value of discontinuous land use and airports land use variables. It was assigned to the “Services - Transport and Distribution” final signature since it had a low cardinality and falls within this larger category of transportation services.

The second set of clusters in this stage resulted from partitioning the “First iteration intermediate cluster 3” into the four urban described in Table 2. This choice was motivated by the most separation in the clustergram, second row in Fig. 8, the best Davies-Bouldin score and a high Calinski-Harabasz score. All the resulting clusters from this step are the final “Urban” clusters and are not processed further.

Lastly for this iteration, the “First iteration intermediate cluster 0” is partitioned into nine clusters. This value was chosen since it identified areas with different functional usages, had good separation in the clustergram, third row in Fig. 8, and average to high cluster metric scores. Similarly to the clustering at the previous stage, lower values of K resulted in many outliers while the larger ones broke down the clusters based on spatial size. The nine clusters are:

- Cluster 0 is the final “Residential - Lower density” cluster.
- Cluster 1 is the “Second iteration intermediate cluster 1” or “SII Cluster 1” in the cluster history graph and is processed further, since it contains a number of distinct functional areas.
- Cluster 2 is the final “Industrial - construction areas” cluster.
- Clusters 3 and 4 represent the “Second iteration intermediate clusters 3/4". Cluster 3 and 4 were grouped together due to both representing industrial areas, assigned due to high values of industrial land use variables, with different magnitudes and sizes. This cluster is processed further in the next iteration.
- Cluster 5 clusters represent port areas, as evidenced by the high value of the port areas variable, while Cluster 7 represents railway stations with high values for the road tracks and associated land and transportation workers variables. These two clusters are combined and assigned to the same final cluster - “Services- transport and distribution hubs” cluster, since they have a small combined cardinality (6,500) and fall within the broader transport functional usage umbrella.
- Clusters 6 and 8 are assigned to the “Outliers” since they delineate areas with high estuaries and marshes, which are not relevant to the functional designation.

**Fig. 9:**
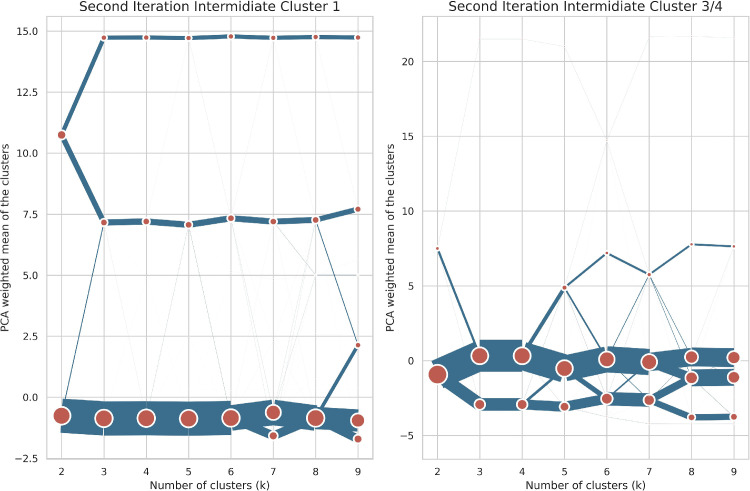
Clustergrams for the final iteration of clustering.

A final 3rd level clustering was chosen for “Second iteration intermediate clusters 3/4" and “Second iteration intermediate cluster 1” clusters, since they covered more than 3 million tessellation cells and potentially different functional areas on the map. The clustergrams associated with this iteration are shown in Fig. 9.

Each row shows the relative separation and size of clusters for different values of K, for specific clusters from the first clustering iteration. The position of the clusters on the Y axis is based on the mean weighted first principle component of the dataset.

The “Second iteration intermediate clusters 3/4" was split into two clusters - the “industrial- commercial areas” and “Industrial - manufacturing” areas. Similarly to the other clusters all possible values were explored, however values less than seven resulted in separating lots of the data with outliers and more than seven did more identify clusters with clear differences in functional usage.

The “Second iteration intermediate cluster 1” was separated into seven clusters. This choice was supported by good separation on the clustergram, a high Calinski-Harabasz score, and the best Davies-Bouldin score. The seven clusters were identified as:

- Cluster 0 of this specific partition represents the final “Services- Mixed - Low Density” cluster.
- Cluster 3 is the final “Services - Transport and distribution” final cluster.
- Cluster 2 is the “Services - Leisure and culture” final cluster”.
- Clusters 1,4 and five represent four types of leisure hotspots with relatively few tessellation cells between them (less than 6,000) - golf ranges, archery ranges and others. All of these clusters were grouped under the “Services - Leisure and culture” final cluster.
- Similarly Cluster 6 has a high number of transport and distribution workers within, but a small number of tessellation cells of 2,300 and therefore was assigned to the much larger “Services - Transport and distribution”

### 2.4 Cluster naming

The last stage of the analysis was the naming of the clusters. The naming scheme and descriptions aim to capture the core distinctions between clusters. The naming of the clusters followed a standardised approach. The first step of the process was to attach to each cluster a family class. For all clusters with the exception of “Residential - Low-density” the classes were chosen based on a parent cluster in the hierarchy. The class names themselves are based on the data distribution differences between the clusters. Residential and Urban were chosen from the first level clustering and Services and Industrial were chosen from the second level clustering. In order to distinguish clusters within the same family, each cluster is further assigned qualifiers based on differences in the distribution of the data within the class. For example, the qualifier “Well-served” was attached to a “Residential” cluster since it had a relatively higher level of public service workers than other clusters in the residential family. A dictionary of the names and qualifiers used, is presented in the Tables 3 and 4 below.

**Table 3:** Cluster classes.

Cluster class	Assignment logic
Urban	First level parent cluster has a high number of employment, cultural, areas, amenities and continuous urban development
Residential	First level parent cluster has a high number of population, public services and discontinuous urban development
Industrial	Second level parent cluster has a high number of industrial and commercial areas
Services	Second level parent cluster has a high number of people working in transportation, distribution, restaurants and a high number of amenities and cultural areas.

The table shows the logic for assigning cluster classes, which are a part of the cluster names. The first column shows the class assigned to one of the final clusters, while the second column describes the assignment logic.

**Table 4:** Cluster qualifiers.

Cluster qualifier	Assignment logic
Well-served	Relatively high public services within the class
Mixed-use	The area has a relatively equal proportions of residential places and workplaces
Low/High density, amenities, cultural areas	Higher/Lower number of population, highlights and amenities

The table shows the logic for assigning cluster qualifiers, which are a part of the cluster names. The first column shows the class assigned to one of the final clusters, while the second column describes the assignment logic.

## CRediT Author Statement

**Krasen Samardzhiev:** Conceptualization, Methodology, Software, Formal analysis, Writing – review & editing; **Martin Fleischmann:** Conceptualization, Methodology, Software, Data curation, Writing – review & editing; **Daniel Arribas-Bel:** Conceptualization, Methodology, Writing – review & editing; **Alessia Calafiore:** Conceptualization, Methodology, Writing – review & editing; **Francisco Rowe:** Conceptualization, Methodology, Writing – review & editing.

## Declaration of Competing Interest

The authors declare that they have no known competing financial interests or personal relationships that could have appeared to influence the work reported in this paper.

## Data Availability

Functional signatures (Original data) (figshare).
